# Supplementary material for: GlobalUsefulNativeTrees, a database documenting 14,014 tree species, supports synergies between biodiversity recovery and local livelihoods in landscape restoration
Source: Sci Rep. 2023 Aug 3;13:12640. doi: 10.1038/s41598-023-39552-1 (PMC10400654; doi:10.1038/s41598-023-39552-1)

Supplementary Figures

Supplementary Fig 1. Subcontinental species richness for GlobalUsefulNativeTrees across different use categories**.** Codes and colour scheme is the same as Fig 1 in the main text, except not to assign the subcontinental colour to a country if that country had no species. Country boundaries added from Natural Earth 1:110 million. Best seen with magnification ≥ 200%.

Supplementary Fig 2. Subcontinental patterns of endemism for GlobalUsefulNativeTrees. Codes and colour scheme is the same as Fig 2 in the main text, except not to assign the subcontinental colour to a country if that country had no species. Country boundaries added from Natural Earth 1:110 million. Best seen with magnification ≥ 200%.

Supplementary Fig 3. Subcontinental patterns of threats for GlobalUsefulNativeTrees. Codes and colour scheme is the same as Fig 3 in the main text, except not to assign the subcontinental colour scheme to a country if that country had no species. Country boundaries added from Natural Earth 1:110 million. Best seen with magnification ≥ 200%.

Supplementary Figure 1

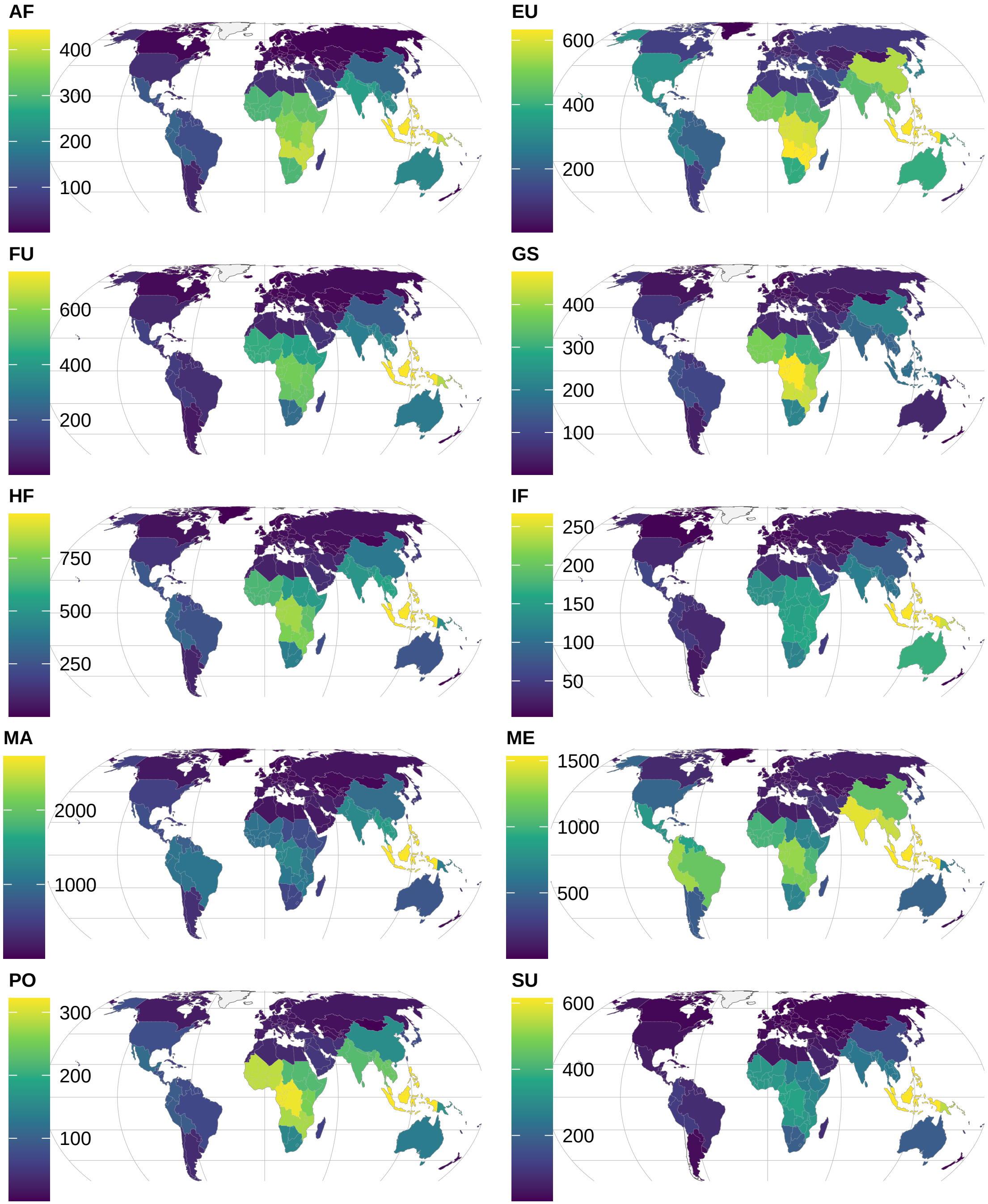

Supplementary Figure 2

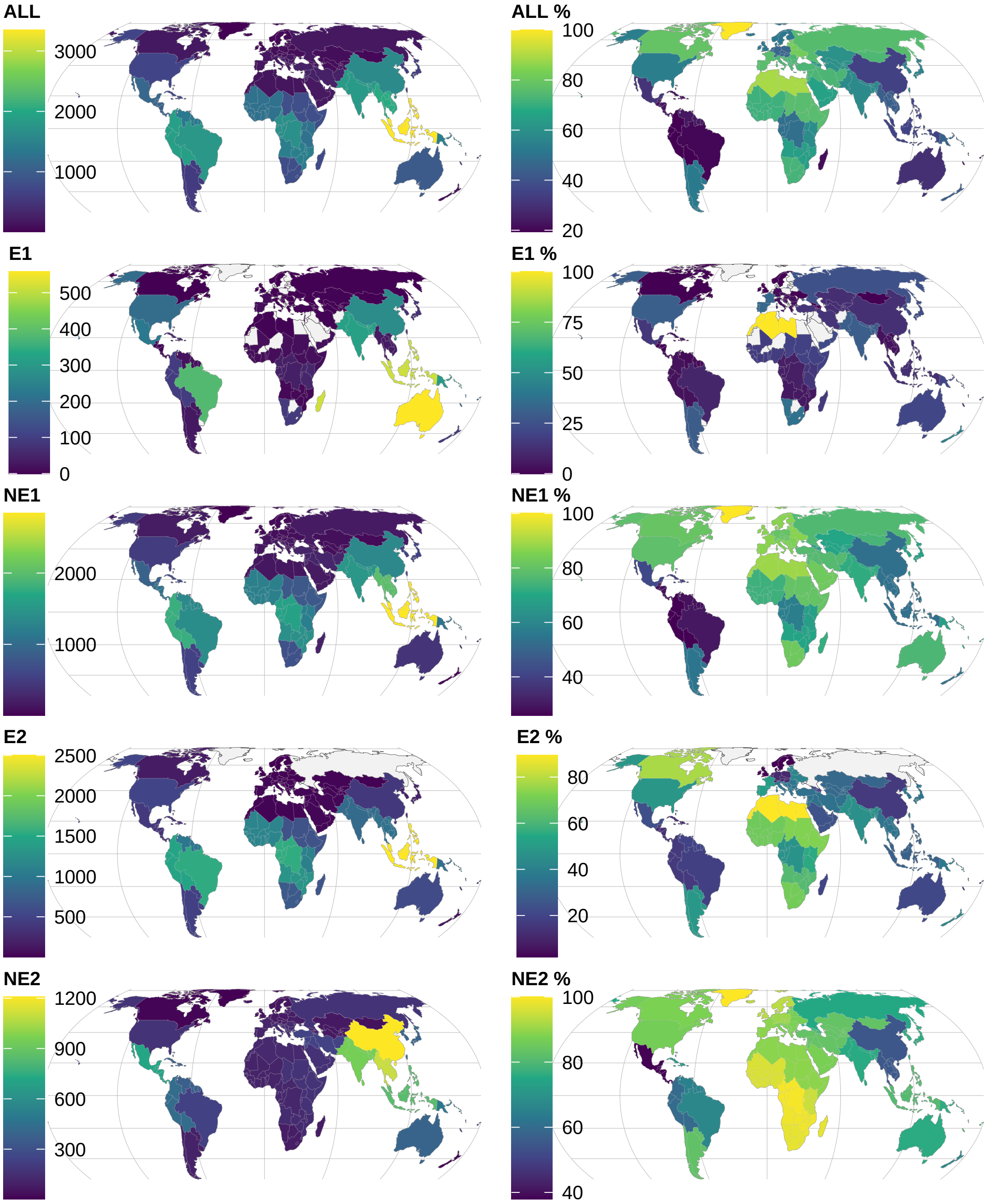

Supplementary Figure 3

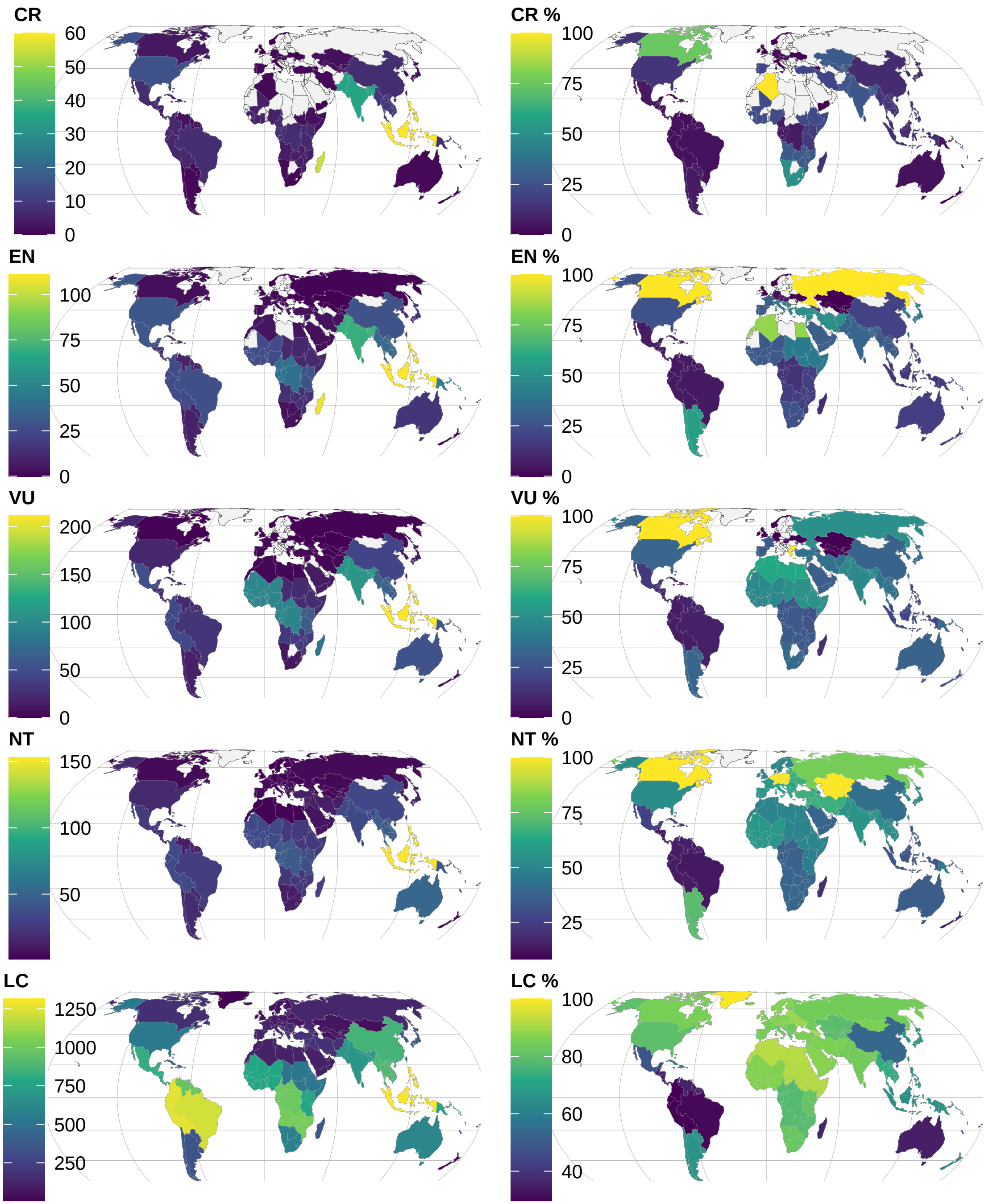

Supplement: Supplementary file 1 — Supplementary Figures. [file 41598_2023_39552_MOESM1_ESM.pdf]
